# Annual risk of falls resulting in emergency department and hospital attendances for older people: an observational study of 781,081 individuals living in Wales (United Kingdom) including deprivation, frailty and dementia diagnoses between 2010 and 2020

**DOI:** 10.1093/ageing/afac176

**Published:** 2022-08-02

**Authors:** Robyn Hollinghurst, Neil Williams, Rebecca Pedrick-Case, Laura North, Sara Long, Richard Fry, Joe Hollinghurst

**Affiliations:** Population Health Data Science, Swansea University Medical School, Swansea University, Swansea, Wales, UK; Care & Repair Cymru, Cardiff, Wales, UK; Population Health Data Science, Swansea University Medical School, Swansea University, Swansea, Wales, UK; Population Health Data Science, Swansea University Medical School, Swansea University, Swansea, Wales, UK; Population Health Data Science, Swansea University Medical School, Swansea University, Swansea, Wales, UK; Population Health Data Science, Swansea University Medical School, Swansea University, Swansea, Wales, UK; Population Health Data Science, Swansea University Medical School, Swansea University, Swansea, Wales, UK

**Keywords:** falls, COVID-19, dementia, frailty, older people

## Abstract

**Background:**

falls are common in older people, but associations between falls, dementia and frailty are relatively unknown. The impact of the COVID-19 pandemic on falls admissions has not been studied.

**Aim:**

to investigate the impact of dementia, frailty, deprivation, previous falls and the differences between years for falls resulting in an emergency department (ED) or hospital admission.

**Study Design:**

longitudinal cross-sectional observational study.

**Setting:**

older people (aged 65+) resident in Wales between 1 January 2010 and 31 December 2020.

**Methods:**

we created a binary (yes/no) indicator for a fall resulting in an attendance to an ED, hospital or both, per person, per year. We analysed the outcomes using multilevel logistic and multinomial models.

**Results:**

we analysed a total of 5,141,244 person years of data from 781,081 individuals. Fall admission rates were highest in 2012 (4.27%) and lowest in 2020 (4.27%). We found an increased odds ratio (OR [95% confidence interval]) of a fall admission for age (1.05 [1.05, 1.05] per year of age), people with dementia (2.03 [2.00, 2.06]) and people who had a previous fall (2.55 [2.51, 2.60]). Compared with fit individuals, those with frailty had ORs of 1.60 [1.58, 1.62], 2.24 [2.21, 2.28] and 2.94 [2.89, 3.00] for mild, moderate and severe frailty respectively. Reduced odds were observed for males (0.73 [0.73, 0.74]) and less deprived areas; most deprived compared with least OR 0.75 [0.74, 0.76].

**Conclusions:**

falls prevention should be targeted to those at highest risk, and investigations into the reduction in admissions in 2020 is warranted.

## Key Points

- Reduction in falls admissions during the COVID-19 pandemic.
- Increased risk of falls resulting in an emergency department or hospital admission for people with dementia.
- Frailty increases falls risk.

## Introduction

Falls in older people is a critical area that has a substantial impact on the individual, their family, health services and society. Falls present a significant economic burden, costing the National Health Service (NHS) in the United Kingdom more than £2.3billion per year [1, 2]. Direct costs from fall-related injuries are estimated to be 0.1% of all healthcare expenditures in the United States and 1.5% in European countries [3].

The frequency of falls increases with age and frailty [4] with people aged 65 or older have the highest risk of falling, with 30% of people aged 65+ and 50% of people aged 80+ falling at least once a year [5]. Previous falls often result in an increased chance of another fall; after a first fall, people have a 66% chance of having another fall within a year [6, 7]. People who have a gait or balance problem are at higher risk of future falls [8]. Dementia is a condition that results in cognition deficits, gait impairments and postural control issues and can contribute to the increased chance of falls [9]. Medications used to treat dementia-related symptoms were identified as increasing falls risk [1]. However, there has been limited research of the direct impact of dementia and the risk of falls [10]. This is despite the annual incidence of falls in older people with dementia being around 70–80%, approximately twice the incidence of falls in cognitively intact older people [11, 12]. Furthermore, current falls prevention approaches are poorly suited to people with dementia [13].

COVID-19 has adversely impacted the number of older people accessing healthcare services. Studies in the United States indicate a 45.4% reduction in hospital admissions amongst older adults in 2020 compared with 2019 [14]. Similarly, a study in Germany showed a 21% reduction in admissions for cardiovascular events for individuals aged 60+ [15].

In this study, we linked administrative and electronic health records (EHRs) to investigate risk factors for fall admissions to hospitals and emergency departments (EDs). We compared the impact and difference in falls risk for people with dementia and those who had a fall in the previous year. We included age, frailty, deprivation and gender as covariates. We analysed falls admissions to hospital and EDs independently and jointly to identify if our risk factors changed depending on the admission type. We also investigated differences in falls admissions in hospitals and EDs between 2020 and previous years to investigate the impact of COVID-19.

## Methods

### Study design

We used longitudinal anonymised EHRs and administrative data to conduct a cross-sectional cohort study [16].

### Data sources

Our cohort was created using data held within the Secure Anonymised Information Linkage (SAIL) Databank [17–19].

In this study we used the following datasets: the Welsh Demographic Service Dataset (WDSD), the Patient Episode Database for Wales (PEDW), the Emergency Department Data Set (EDDS) and the Welsh Longitudinal General Practice dataset (WLGP). We used the WDSD for demographic and residency information for each individual. We used PEDW and EDDS for details on emergency hospital admissions and ED attendances, respectively. The WLGP was used to determine general practice registration history and to calculate the electronic Frailty Index (eFI).

### Setting

Individuals in Wales aged 65+ years who were registered with a general practice submitting data to the SAIL Databank. SAIL currently receives data from 80% of general practices in Wales [20]. The study covered data from 1 January 2010 to 31 December 2020.

### Participants

Individuals who had a full year of residential history during each of the study years were included. For example, to be included in the study year 2010–11 a participant was recorded as a Welsh resident from 1 January 2010 to 31 December 2010. In total, we analysed data for 781,081 individuals.

### Dataset design

The dataset contained 5,141,244 observations. Each year of study was created independently to ensure individuals were aged 65+ at the start of the study year. Individuals were only included in the study year if a full year of residential information was available. This ensured individuals were recorded as being resident in Wales for the duration of the study year and not in their final year of life.

### Variables

#### Primary outcome—falls

The primary outcome was a fall related attendance at an emergency department (ED) or admission to hospital. We created a binary variable to indicate if an individual had a fall resulting in an attendance to either an ED or hospital for use in our analysis. If an individual attended the ED and was then admitted to hospital in the same year this was counted as a single event. We have specified the codes used to identify falls in the Supplementary Material, section Falls coding.

#### Secondary outcome—falls in either an ED or hospital

For a secondary analysis we disaggregated the primary outcome to either a fall resulting in an ED attendance or a hospital attendance and analysed the outcomes separately.

#### Tertiary outcome—mutually exclusive falls categories

As a third outcome we created a set of distinct, independent, indicators for each individual with one of the following categories: no falls, falls admissions to hospital only, an admission to ED only or both hospital and ED.

#### Falls in the previous year

We used the same method as the primary outcome to determine if someone had a fall resulting in attendance to either an ED or hospital in the previous year.

#### SAIL dementia e-Cohort

To determine dementia diagnoses we used the SAIL dementia e-Cohort (SDeC) [21]. The SDeC uses validated code lists to search for diagnoses of dementia in primary and secondary care. Individuals were identified as having a dementia diagnosis if the diagnosis preceded the start of the study year. The SDeC also identifies the following subtypes of dementia: Alzheimer’s, dementia with Lewy bodies, vascular dementia and frontotemporal dementia.

#### Electronic Frailty Index

The eFI assigns a frailty score to an individual using 36 variables from primary care data including falls, symptoms, signs, diseases, disabilities and abnormal laboratory values, referred to as deficits [22]. The eFI score is the number of deficits present, expressed as an equally weighted proportion of the total. An individual with nine deficits would be assigned an eFI of 9/36 (0.25). The eFI score categorises individuals as: fit (eFI value of 0–0.12), mild (>0.12–0.24), moderate (>0.24–0.36) or severe frailty (>0.36) [23, 24]. We calculated the eFI on the 1 January of each year using a 10-year window of prior data.

#### Welsh Index of Multiple Deprivation

We included the 2014 Welsh Index of Multiple Deprivation (WIMD) quintile as a measure of socioeconomic status [25]. We assigned a WIMD quintile based on an individual’s residence on the 1 January of the study year.

#### Additional variables

Age was calculated at the start of the study year. Gender was fixed for the study year.

### Statistical methods

In our primary analysis the dependent variable was the binary indicator for an ED attendance or hospitalisation following a fall. In the secondary analyses we analysed binary indicators for ED attendance and hospital separately. In the third analysis the outcome was either: no fall, a fall admission to hospital only, an admission to ED only or an admission to both hospital and ED. Dementia diagnosis (yes/no), falls in the previous year (yes/no), gender (male/female), eFI (fit, mild, moderate, severe) and WIMD (1. Most deprived to 5. Least deprived) were included as categorical variables. Age was included as a continuous variable, with a minimum value of 65.

Descriptive statistics were created for each year of study including the number and rate of falls admissions per year. Differences in the proportion of people with subtypes of dementia (Alzheimer’s, dementia with Lewy bodies, vascular dementia and frontotemporal dementia) who had a fall admission in each year of study were tested using chi-squared tests. Univariable and multivariable multilevel logistic regression models were used to calculate odds ratios (ORs) and 95% confidence intervals for the 1st and 2nd analyses. For the 3rd analysis a multilevel multinomial model was used. A logit link was used in the regression analyses and a random intercept term was included for each study year; an individual level random effect was fitted as a sensitivity analysis. Additional sensitivity analyses included: excluding 2020 from the multilevel multivariable model and computing a fixed effects model with an interaction between dementia diagnosis and admission year. The residuals for age and age versus the rate of falls were plotted to test the assumption of a linear predictor in the regression models. Analyses were conducted using R version 4.0.3 and R2MLwiN version 3.05 [26].

## Results

### Descriptive statistics

We analysed 5,141,244 person years of data, with an average of 467,386 individuals per year, see Supplementary Figure S1 for the dataset derivation. Descriptive data for a subset of years for the cohort along with stratifications for those who had a fall admission are provided in Table 1. There was a greater mean age of ~5-years for those who had a fall admission. Among those who had a fall admission, there was a higher percentage of females, moderate and severe frailty, people with dementia, people who had a previous fall admission and people living in the most deprived areas.

**Table 1:** Descriptive statistics for cohort years 2010–11, 2015–16 and 2020–21

	Whole cohort			Individuals who had a fall			Individuals who did not fall		
Cohort Year	2010–11	2015–16	2020–21	2010–11	2015–16	2020–21	2010–11	2015–16	2020–21
***N***	412,458	472,246	509,671	13,851	17,723	15,908	398,607	454,523	493,763
**Falls rate (%)**				3.36%	3.75%	3.12%			
**Mean Age (S.D.)**	75.0 (7.5)	74.7 (7.5)	75.1 (7.3)	79.5 (8.3)	79.2 (8.5)	79.4 (8.4)	74.8 (7.4)	74.6 (7.4)	74.9 (7.3)
**Sex—male**	185,507 (45%)	217,123 (46%)	235,934 (46%)	4,476 (32%)	6,144 (35%)	5,659 (36%)	181,031 (45%)	210,979 (46%)	230,275 (47%)
**Sex—female**	226,951 (55%)	255,123 (54%)	273,737 (54%)	9,375 (68%)	11,579 (65%)	10,249 (64%)	217,576 (55%)	243,544 (54%)	263,488 (53%)
**Dementia**	16,279 (4%)	19,118 (4%)	20,609 (4%)	1,879 (14%)	2,407 (14%)	2,268 (14%)	14,400 (4%)	16,711 (4%)	18,341 (4%)
**eFI—fit**	197,933 (48%)	222,008 (47%)	255,980 (50%)	3,702 (27%)	4,416 (25%)	4,228 (27%)	194,231 (49%)	217,592 (48%)	251,752 (51%)
**eFI—mild**	152,396 (37%)	174,448 (37%)	181,881 (36%)	5,624 (41%)	7,114 (40%)	6,428 (40%)	146,772 (37%)	167,334 (37%)	175,453 (36%)
**eFI—moderate**	51,148 (12%)	61,498 (13%)	58,961 (12%)	3,402 (25%)	4,518 (25%)	3,850 (24%)	47,746 (12%)	56,980 (13%)	55,111 (11%)
**eFI—severe**	10,981 (3%)	14,292 (3%)	12,849 (3%)	1,123 (8%)	1,675 (9%)	1,402 (9%)	9,858 (2%)	12,617 (3%)	11,447 (2%)
**WIMD—1. Most deprived**	68,970 (17%)	77,538 (16%)	81,217 (16%)	2,810 (20%)	3,898 (22%)	3,340 (21%)	66,160 (17%)	73,640 (16%)	77,877 (16%)
**2**	82,627 (20%)	91,388 (19%)	98,885 (19%)	2,804 (20%)	4,064 (23%)	3,723 (23%)	79,823 (20%)	87,324 (19%)	95,162 (19%)
**3**	85,978 (21%)	99,928 (21%)	107,486 (21%)	2,533 (18%)	3,286 (19%)	3,110 (20%)	83,445 (21%)	96,642 (21%)	104,376 (21%)
**4**	80,809 (20%)	93,309 (20%)	101,939 (20%)	2,341 (17%)	2,768 (16%)	2,492 (16%)	78,468 (20%)	90,541 (20%)	99,447 (20%)
**5.Least deprived**	94,074 (23%)	110,083 (23%)	120,144 (24%)	3,363 (24%)	3,707 (21%)	3,243 (20%)	90,711 (23%)	106,376 (23%)	116,901 (24%)
**Previous falls**	7,829 (2%)	14,650 (3%)	15,505 (3%)	995 (7%)	1,941 (11%)	1,821 (11%)	6,834 (2%)	12,709 (3%)	13,684 (3%)

**Figure 1:**
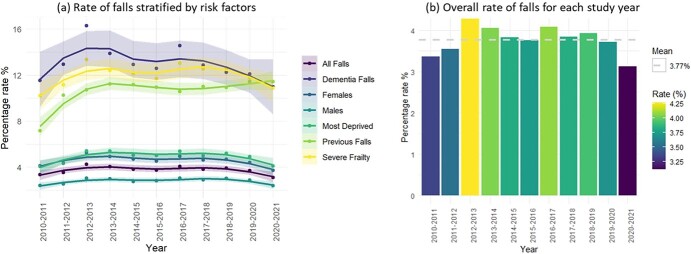
(a) Comparison of the rate of falls per year for: the whole cohort, individuals with a dementia diagnosis, gender (male/female), the most deprived WIMD quintile, individuals who had previous fall and individuals with severe frailty. (b) The overall rate of falls for each study year with the mean rate included.

The rate of falls admissions per year is displayed in Figure 1a, which shows a comparison of falls admissions between people with dementia, people who had a previous fall admission, people with severe frailty, gender and deprivation. The comparison shows the rate of falls admissions was the highest for those with dementia followed by people with severe frailty and those who had a previous fall admission, with the rates being significantly greater than other factors. The proportion of people with different subtypes of dementia who had a fall admission was statistically significantly different in all study years except 2010–11, with generally lower rates in those diagnosed with Alzheimer’s and generally higher rates in people with dementia with Lewy bodies, frontotemporal dementia and vascular dementia (Supplementary Table S1, Supplementary Figure S2). Figure 1b displays the overall rate of falls admissions for each study year along with the mean rate (3.8%) across the entire study.

### Analysis of falls resulting in an ED or hospital admission

#### Multilevel logistic regression models

The univariable and multivariable multilevel logistic regression model results are provided in Tables 2 and 3, respectively. The models indicated increased odds of a fall admission for people with dementia, people with severe frailty and people who had a previous fall. This model suggests that the highest odds of a fall-related admission are for those with severe frailty. For dementia and severe frailty, the increased odds were greater for falls resulting in hospital admissions compared with ED admissions. However, this was the reverse for individuals who had a previous admission for a fall. Decreased odds of a fall resulting in an emergency or hospital admission were seen for males compared with females and for less deprived areas. The ORs for these factors were similar, regardless of falls resulting in hospital admissions, ED admissions or both combined.

**Table 2:** Univariable models for falls resulting in hospital and ED admissions

ORs (95% Confidence interval)	Hospital or ED	Hospital	ED
**Intercept**	0.04 (0.04, 0.04)	0.02 (0.02, 0.02)	0.03 (0.03, 0.03)
**Age**	1.07 (1.07, 1.07)	1.10 (1.10, 1.10)	1.06 (1.06, 1.07)
**Gender (baseline female)**			
**Male**	0.60 (0.60, 0.61)	0.58 (0.57, 0.58)	0.6 (0.60, 0.61)
**Dementia (baseline no dementia diagnosis)**			
**Dementia**	4.28 (4.22, 4.34)	5.32 (5.22, 5.42)	3.79 (3.73, 3.86)
**Frailty (baseline fit)**			
**eFI-Mild**	2.08 (2.06, 2.10)	2.40 (2.36, 2.44)	1.99 (1.96, 2.02)
**eFI-Moderate**	3.91 (3.86, 3.96)	5.14 (5.04, 5.25)	3.51 (3.46, 3.56)
**eFI-Severe**	6.60 (6.48, 6.72)	9.45 (9.21, 9.69)	5.55 (5.43, 5.67)
**Deprivation (baseline 1. Most deprived)**			
**2.**	0.89 (0.87, 0.90)	0.97 (0.95, 0.99)	0.85 (0.84, 0.87)
**3.**	0.68 (0.67, 0.69)	0.87 (0.85, 0.89)	0.58 (0.57, 0.58)
**4.**	0.59 (0.58, 0.60)	0.83 (0.81, 0.85)	0.47 (0.46, 0.48)
**5. Least deprived**	0.70 (0.69, 0.71)	0.80 (0.78, 0.81)	0.65 (0.64, 0.66)
**Previous fall (baseline no previous fall)**			
**Previous fall**	4.33 (4.27, 4.40)	4.17 (4.08, 4.27)	4.60 (4.52, 4.68)
**Random effects for the null models (Year)**			
**Intercept variance (Standard Error)**	0.0076 (0.0032)	0.0020 (0.0009)	0.0158 (0.0068)

**Table 3:** Multivariable models for falls resulting in hospital and ED admissions

ORs (95% Confidence Interval)	Hospital or ED	Hospital	ED
**Intercept**	0.001 (0.001, 0.001)	0.0001 (0.0001, 0.0001)	0.0017 (0.0016, 0.0019)
**Age**	1.05 (1.05, 1.05)	1.07 (1.07, 1.07)	1.04 (1.04, 1.04)
**Gender (baseline female)**			
**Male**	0.73 (0.73, 0.74)	0.75 (0.74, 0.76)	0.73 (0.72, 0.73)
**Dementia (baseline no dementia diagnosis)**			
**Dementia**	2.03 (2.00, 2.06)	2.12 (2.08, 2.17)	1.90 (1.86, 1.93)
**Frailty (baseline fit)**			
**eFI-Mild**	1.60 (1.58, 1.62)	1.70 (1.66, 1.73)	1.56 (1.54, 1.59)
**eFI-Moderate**	2.24 (2.21, 2.28)	2.51 (2.46, 2.57)	2.11 (2.07, 2.14)
**eFI-Severe**	2.94 (2.89, 3.00)	3.42 (3.33, 3.52)	2.62 (2.56, 2.68)
**Deprivation (baseline 1. Most deprived)**			
**2.**	0.89 (0.88, 0.90)	0.97 (0.94, 0.99)	0.86 (0.85, 0.87)
**3.**	0.70 (0.69, 0.71)	0.91 (0.89, 0.93)	0.60 (0.59, 0.61)
**4.**	0.63 (0.62, 0.64)	0.88 (0.86, 0.90)	0.50 (0.49, 0.51)
**5. Least deprived**	0.75 (0.74, 0.76)	0.85 (0.83, 0.87)	0.70 (0.68, 0.71)
**Previous fall (baseline no previous fall)**			
**Previous fall**	2.55 (2.51, 2.60)	2.13 (2.08, 2.18)	2.82 (2.77, 2.87)
**Random effects (Year)**			
**Intercept variance (Standard Error)**	0.0064 (0.0028)	0.0014 (0.0007)	0.0143 (0.0061)

The residuals for age from a randomly sampled cohort suggested no additional non-linear terms needed to be added to the model (Supplementary Tables S2 and S3, Supplementary Figures S3–S5). The random intercept residuals for the null (intercept only) and multivariable models are presented in Supplementary Figure S6. The 2010–11, 2011–12 and 2020–21 years showed a statistically significant reduction in falls compared with other years. The model with an individual-level effect is presented in Supplementary Table S4.

#### Multinomial model

The results from the multilevel multinomial model are presented in Table 4. In the model, age, people with a dementia diagnosis, increased frailty severity and previous falls were all associated with a higher odds of a fall admission, irrespective of admission type. Males had a reduced odds of a fall admission for all outcomes. There was a reduced odds of an ED admission for lower levels of deprivation but an increased odds of a hospital admission.

**Table 4:** Multilevel multinomial model for falls admissions. Independent outcome categories are: no falls (baseline), emergency department falls admissions only, hospital admissions for falls only and both an emergency department and hospital admission

Multinomial model (baseline: no falls admission)			
ORs (95% confidence interval)	ED only	Hospital only	ED and Hospital
**Intercept**	0.0027 (0.0024, 0.0029)	0 (0, 0)	0 (0, 0)
**Age**	1.03 (1.03, 1.03)	1.07 (1.07, 1.07)	1.07 (1.07, 1.07)
**Gender (baseline female)**			
**Male**	0.73 (0.72, 0.74)	0.77 (0.76, 0.79)	0.71 (0.69, 0.72)
**Dementia (baseline no dementia diagnosis)**			
**Dementia**	1.82 (1.79, 1.86)	2.17 (2.11, 2.23)	2.18 (2.12, 2.25)
**Frailty (baseline fit)**			
**eFI—mild**	1.55 (1.53, 1.58)	1.72 (1.68, 1.76)	1.70 (1.65, 1.75)
**eFI—moderate**	2.06 (2.03, 2.10)	2.64 (2.57, 2.71)	2.43 (2.35, 2.52)
**eFI—severe**	2.52 (2.46, 2.59)	3.72 (3.59, 3.85)	3.21 (3.07, 3.35)
**Deprivation (baseline 1. Most deprived)**			
**2.**	0.85 (0.84, 0.87)	1.04 (1.00, 1.07)	0.88 (0.86, 0.91)
**3.**	0.60 (0.58, 0.61)	1.16 (1.12, 1.19)	0.62 (0.60, 0.64)
**4.**	0.50 (0.48, 0.50)	1.20 (1.16, 1.23)	0.52 (0.50, 0.54)
**5. Least deprived**	0.71 (0.69, 0.72)	1.01 (0.98, 1.05)	0.67 (0.64, 0.69)
**Previous fall (baseline no previous fall)**			
**Previous fall**	2.73 (2.67, 2.79)	1.68 (1.62, 1.73)	3.13 (3.03, 3.23)
The random part estimates at the study year level:			
Intercept variance	0.02 (0.01)	0.01 (0)	0.01 (0)
Covariance:	No falls, ED and Hospital falls	No falls, Hospital falls	Hospital falls, ED and Hospital falls
	0.01 (0)	−0.01 (0)	0 (0)

Although there was an increased odds of admission for people with dementia, increased frailty and previous falls, the ORs varied between outcomes. For people with dementia the odds of hospital admission only was 2.17 (2.11, 2.23), compared with 1.82 (1.79, 1.86) for ED admissions only. Similarly, for severe frailty the odds of hospital admission only was 3.72 (3.59, 3.85), compared with 2.52 (2.46, 2.59) for ED admissions only. For previous falls the odds of hospital admission only was 1.68 (1.62, 1.73), compared with 2.73 (2.67, 2.79) for ED admissions only and 3.13 (3.03, 3.23) for both ED and hospital admissions.

### Sensitivity analyses

Excluding 2020 showed no significant differences in the fixed effect estimates, but halved the intercept variance (Supplementary Table S5). The interaction model showed a small but significant reduction in the odds of a fall admission for 2020 compared with 2010 and for those with a dementia diagnosis in 2020 with ORs of 0.998 (0.997, 0.998) and 0.994 (0.990, 0.998), respectively (Supplementary Table S6).

## Discussion

The study showed age, gender, frailty, deprivation, dementia and previous falls are all risk factors associated with a fall resulting in admissions to hospital and EDs. The mean age for an older individual (65+) who had a fall admission was 79; 5 years older than those that did not have a fall. Increased age is associated with sarcopenia that causes slower physical performance and reflexes due to reduced muscle mass, which can result in severe falls and subsequent hospitalisation [27, 28]. The percentage of females that had a fall admission was greater than males. This may be related to levels of physical activity, strength, bone mass and willingness to seek medical attention [17].

The results indicated dementia is an important risk factor associated with falls admissions. The rate of falls admissions for individuals with dementia was considerably higher than the total cohort (e.g. 12.59% compared with 3.75% in 2015–16). The regression models corroborated this with an increased odds of a fall admission (OR [95% confidence interval]) for people with dementia (2.03 [2.00, 2.06]). Research has investigated cognition and gait and balance problems as risks associated with falls, but little research has specifically linked them to dementia [10]. We also found significant differences in the proportion of people who had a fall admission depending on dementia subtypes; this could be associated with differing levels of cognitive and physical ability.

Severe frailty and previous falls admissions are important risk factors associated with falls. This can be seen by the increased odds for those with severe frailty compared with those defined as fit; OR 2.94 [2.89, 3.00], and those with previous falls admissions compared with without; OR 2.55 [2.51, 2.60]. The descriptive data for individuals who had a fall showed 2,268 (14%) individuals with dementia had a fall in 2020–21, compared with 1,402 (9%) individuals with severe frailty and 1,821 (11%) who had a previous fall. This suggests falls prevention approaches should be targeting people with dementia as well as severe frailty and a history of falls.

In the multinomial model we found a difference in the odds of a fall for deprivation quintiles depending on the attendance type. This could indicate a disparity in attending different services depending on deprivation level and the availability of services. Specifically, ED attendances had a reduced odds for lower levels of deprivation (more affluent areas), but generally increased odds for hospital attendances. This could also relate to the severity of falls, with those from more deprived backgrounds only seeking services in an emergency.

Another result of interest was found when considering the overall rate of falls resulting in admissions to hospital and EDs per year. The mean rate for falls admissions was 3.8%, but in 2020–21 the rate of falls was 3.1%, the lowest across the whole study period. This could be due to the COVID-19 pandemic causing a change in behaviour in older adults. For example, older adults ‘shielding’ and not wanting to leave home, or a reluctance to attend hospital or an ED due to fear of contracting the virus. This could be problematic if someone had a fall and did not seek help as if left untreated this could have a severe impact on the individual and subsequently future hospital attendances.

The comparison of the rate of falls admissions stratified by the different risk factors indicated there was a decline in the levels of admissions for older people in 2020; the rate of falls for 2020–21 decreased from the previous year for each risk factor apart from individuals who had a previous fall admission. Again, this decline in falls admissions could be a result of the impact of COVID-19 and the reluctance to be in a hospital environment. However, the rate for individuals who had a previous fall remained constant in 2020–21 compared with the previous year. This could be due to people who were admitted to hospital previously having a more serious injury or were less hesitant to be readmitted.

### Limitations

Our logistic regression analyses did not take in to account the time at risk, and the exclusion of individuals without an entire year of data may bias our coefficient estimates. Specifically, we may have underestimated the odds of a fall in those who are in their final year of life. Due to data availability we only included individuals who were registered with general practices that contribute data to the SAIL databank. We were also limited to data on falls admissions to an ED or hospital, meaning our study focussed on severe falls. We also note that we have not investigated the sensitivity or specificity of falls coding in secondary care and how coding priorities may have changed during the pandemic. Primary care dementia diagnoses are contained in both the eFI calculation as part of the memory and cognitive problems deficit and the SDeC that may have caused collinearity in the multivariable models. However, the eFI provides an overall frailty category, whereas the SDeC is dementia specific, provides information on dementia sub-types and has greater coverage for dementia diagnoses with a larger number of primary and secondary care codes. Due to data availability we were unable to include the severity of dementia or previous falls in our analysis that may provide a more granular effect size estimate.

### Strengths

We were able to create a large dataset for analysis, providing substantial statistical power. We also included data from 2020, which provides valuable insight into the impact of COVID-19 on hospital and emergency attendances for older people. In addition, we were able to link across datasets to provide details on demographics, dementia diagnoses, frailty and previous falls.

## Conclusion

Our study showed an increased odds of a fall resulting in an admission to an ED or hospital for people who had a previous fall, those with frailty and those with dementia. For those with a dementia diagnosis we found generally higher rates of falls in people diagnosed with dementia with Lewy bodies, frontotemporal dementia and vascular dementia. Results also highlighted differences in attendance types depending on deprivation status, with people living in more deprived areas more likely to attend EDs for falls than be admitted to hospital. We also found a significant decrease in the number of attendances in 2020 compared with previous years, highlighting the need for further research into healthcare utilisation during the COVID-19 pandemic and the subsequent consequences.

## Supplementary Material

aa-21-1788-File002_afac176
