# Supplementary material for: Annual risk of falls resulting in emergency department and hospital attendances for older people: an observational study of 781,081 individuals living in Wales (United Kingdom) including deprivation, frailty and dementia diagnoses between 2010 and 2020
Source: Age Ageing. 2022 Aug 2;51(8):afac176. doi: 10.1093/ageing/afac176 (PMC9356534; doi:10.1093/ageing/afac176)
Supplement: aa-21-1788-File002_afac176 [file aa-21-1788-file002_afac176.docx]

Annual risk of falls resulting in emergency department and hospital attendances for older people: An observational study of 781,081 individuals living in Wales (United Kingdom) including deprivation, frailty, and dementia diagnoses between 2010-2020

**SUPPLEMENTARY DATA:**

- Figure S1. Dataset derivation and limitations LSOA: Lower-layer Super Output Area.
- Table S1. The rate and number of falls per study year for dementia and subtypes of dementia. Chi-squared tests were used to test for significant differences in the proportions of falls between dementia subtypes for each study year.
- Figure S2. The rate of people who had a fall per year, stratified by subtypes of dementia.
- Table S2. Randomly sampled dataset for the age residual analysis
- Table S3 Univariable and multivariable models for the age residual analysis
- Figure S3. Quantile residuals for age from the univariable logistic regression model
- Figure S4. Quantile residuals for age from the multivariable logistic regression model.
- Figure S5. The proportion of people who had a fall versus age. A linear regression line has been fitted to the plot.
- Figure S6. The study year residuals for the null (intercept only) and multivariate multilevel logistic regression models. (a) The null model residuals ordered by the study year. (b) The null model ordered by the residual value. (c) The multivariate model residuals ordered by the study year. (d) The multivariate model ordered by the residual values.
- Table S4. Multilevel logistic regression models for falls admissions resulting in a hospital or emergency department admission. The models include a random effect at the person level.
- Table S5. Multilevel multivariable logistic regression model with data from 2020 removed. The model includes a random intercept term for each year.
- Table S6. Logistic regression model with an interaction term between year and dementia diagnosis included.
- Falls coding

Figure S1. Dataset derivation and limitations LSOA: Lower-layer Super Output Area.


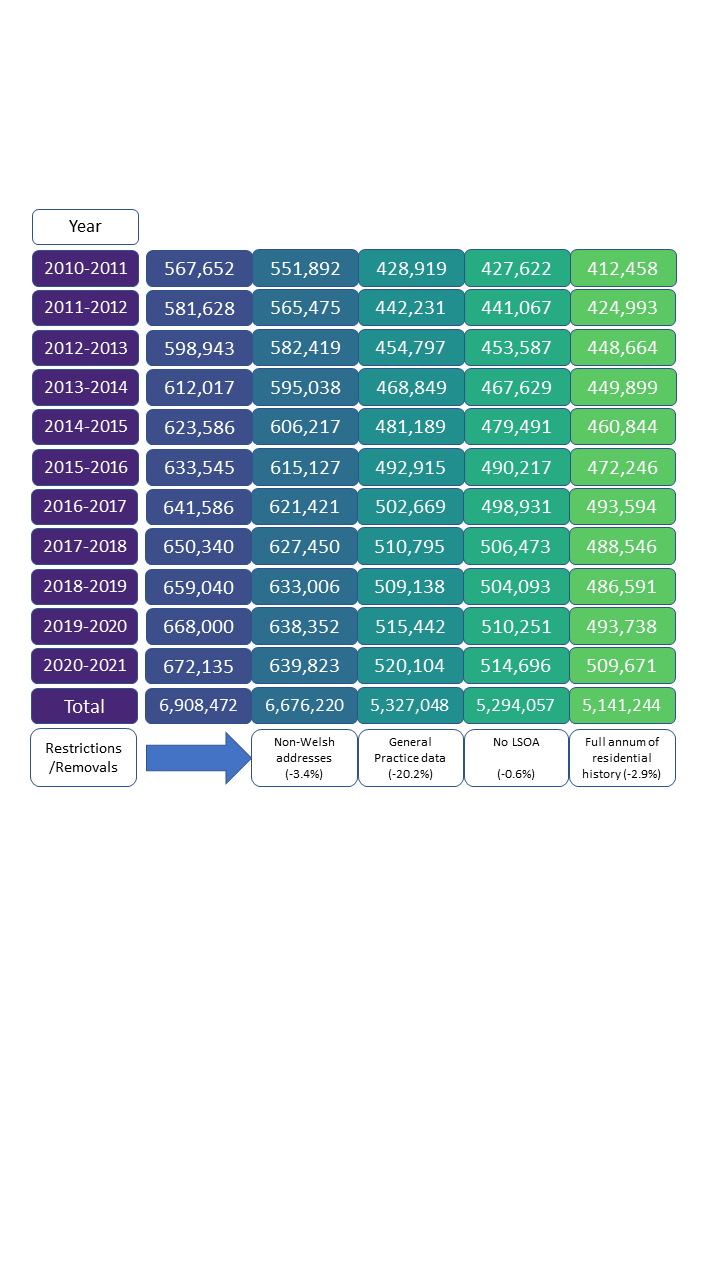


**Table S1. The rate and number of falls per study year for dementia and subtypes of dementia. Chi-squared tests were used to test for significant differences in the proportions of falls between dementia subtypes for each study year.**

| Year | Dementia | Alzheimers | Vascular | Fronto-temporal | Lewy Bodies | Chi-squared | degrees of freedom | p-value |
| --- | --- | --- | --- | --- | --- | --- | --- | --- |
| 2010-2011 | 11.54% (1879/16279) | 10.88% (823/7565) | 12.43% (730/5872) | 14.39% (19/132) | 10.58% (20/189) | 9.02 | 4 | 0.061 |
| 2011-2012 | 12.96% (2190/16897) | 11.96% (938/7843) | 14.43% (889/6161) | 11.49% (17/148) | 14.1% (33/234) | 19.21 | 4 | 0.001 |
| 2012-2013 | 16.3% (3208/19681) | 15.13% (1399/9246) | 18.32% (1310/7152) | 20.92% (41/196) | 17.65% (48/272) | 33.31 | 4 | <0.001 |
| 2013-2014 | 13.9% (2487/17897) | 13.25% (1150/8676) | 15.99% (1007/6299) | 20.2% (40/198) | 19.85% (53/267) | 37.05 | 4 | <0.001 |
| 2014-2015 | 12.94% (2418/18692) | 12.02% (1103/9173) | 14.41% (939/6515) | 9.04% (17/188) | 17.03% (54/317) | 26.47 | 4 | <0.001 |
| 2015-2016 | 12.59% (2407/19118) | 11.98% (1145/9560) | 14.47% (944/6524) | 10.71% (21/196) | 15.76% (55/349) | 26.37 | 4 | <0.001 |
| 2016-2017 | 14.58% (3212/22033) | 13.56% (1516/11181) | 16.57% (1254/7568) | 18.06% (41/227) | 15.97% (65/407) | 35.55 | 4 | <0.001 |
| 2017-2018 | 12.88% (2541/19727) | 12.25% (1227/10017) | 14.92% (953/6387) | 14.42% (31/215) | 13.84% (49/354) | 26.33 | 4 | <0.001 |
| 2018-2019 | 12.26% (2359/19238) | 11.67% (1140/9768) | 14.59% (853/5847) | 18.98% (41/216) | 14.78% (51/345) | 40.19 | 4 | <0.001 |
| 2019-2020 | 12.1% (2415/19955) | 11.58% (1155/9978) | 14.19% (805/5673) | 10.9% (23/211) | 16.47% (55/334) | 30.08 | 4 | <0.001 |
| 2020-2021 | 11% (2268/20609) | 10.05% (999/9944) | 12.57% (661/5259) | 9.57% (20/209) | 13.8% (45/326) | 25.54 | 4 | <0.001 |

**Figure S2. The rate of people who had a fall per year, stratified by subtypes of dementia.**


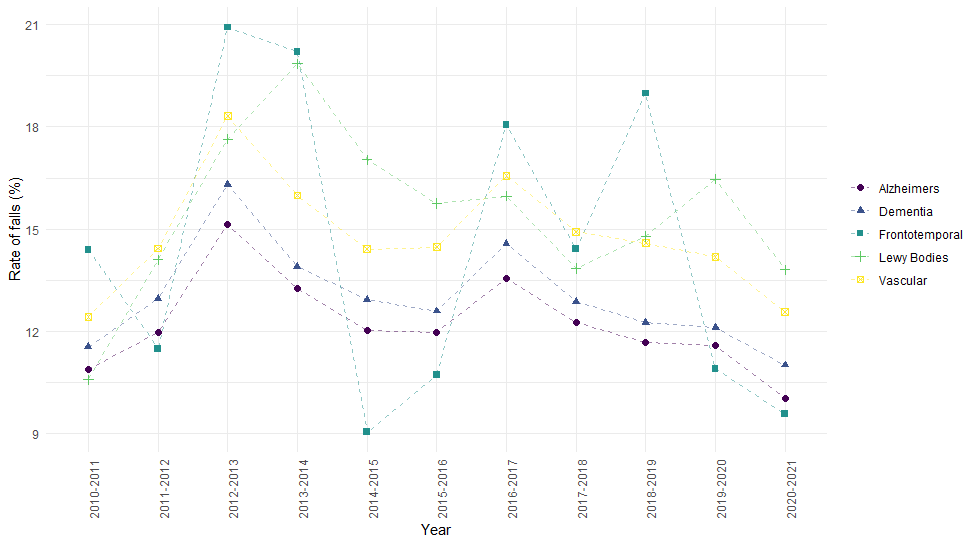


**Additional analysis for the age residuals**

**Table S2. Randomly sampled dataset for the age residual analysis**

|  | Fall admission | |  |
| --- | --- | --- | --- |
|  | No | Yes | Overall |
|  | (N=96290) | (N=3710) | (N=100000) |
| Age |  |  |  |
| Mean (SD) | 74.7 (7.35) | 79.1 (8.39) | 74.9 (7.44) |
| Gender |  |  |  |
| Female | 51544 (53.5%) | 2407 (64.9%) | 53951 (54.0%) |
| Male | 44746 (46.5%) | 1303 (35.1%) | 46049 (46.0%) |
| Dementia |  |  |  |
| No | 92744 (96.3%) | 3187 (85.9%) | 95931 (95.9%) |
| Yes | 3546 (3.7%) | 523 (14.1%) | 4069 (4.1%) |
| eFI (Frailty) |  |  |  |
| Fit | 46993 (48.8%) | 985 (26.5%) | 47978 (48.0%) |
| Mild | 35350 (36.7%) | 1490 (40.2%) | 36840 (36.8%) |
| Moderate | 11463 (11.9%) | 926 (25.0%) | 12389 (12.4%) |
| Severe | 2484 (2.6%) | 309 (8.3%) | 2793 (2.8%) |
| WIMD (2014) |  |  |  |
| 1. Most deprived | 15454 (16.0%) | 813 (21.9%) | 16267 (16.3%) |
| 2 | 18375 (19.1%) | 818 (22.0%) | 19193 (19.2%) |
| 3 | 20402 (21.2%) | 712 (19.2%) | 21114 (21.1%) |
| 4 | 19502 (20.3%) | 567 (15.3%) | 20069 (20.1%) |
| 5. Least deprived | 22557 (23.4%) | 800 (21.6%) | 23357 (23.4%) |
| Previous fall |  |  |  |
| No | 93706 (97.3%) | 3321 (89.5%) | 97027 (97.0%) |
| Yes | 2584 (2.7%) | 389 (10.5%) | 2973 (3.0%) |

**Table S3 Univariable and multivariable models for the age residual analysis**

| **Univariable model** | Coefficient | Standard error | z | p-value |
| --- | --- | --- | --- | --- |
| (Intercept) | -8.668675 | 0.160832 | -53.9 | <2e-16 |
| Age | 0.070446 | 0.002027 | 34.75 | <2e-16 |
|  |  |  |  |  |
| **Multivariable model** | Estimate | Standard error | z | p-value |
| (Intercept) | -6.731992 | 0.175377 | -38.386 | 2.00E-16 |
| Age | 0.044318 | 0.002271 | 19.519 | 2.00E-16 |
| Gender (male) | -0.289787 | 0.035803 | -8.094 | 5.78E-16 |
| Dementia (yes) | 0.733923 | 0.054746 | 13.406 | 2.00E-16 |
| EFI(Mild) | 0.431136 | 0.042981 | 10.031 | 2.00E-16 |
| Moderate | 0.787377 | 0.051227 | 15.37 | 2.00E-16 |
| Severe | 0.961884 | 0.07485 | 12.851 | 2.00E-16 |
| WIMD Quintiles: 2 | -0.150217 | 0.051696 | -2.906 | 0.00366 |
| 3 | -0.380813 | 0.053394 | -7.132 | 9.88E-13 |
| 4 | -0.529321 | 0.056721 | -9.332 | 2.00E-16 |
| 5 | -0.333031 | 0.051966 | -6.409 | 1.47E-10 |
| Previous fall (Yes) | 0.914414 | 0.060148 | 15.203 | 2.00E-16 |

**Figure S3. Quantile residuals for age from the univariable logistic regression model**


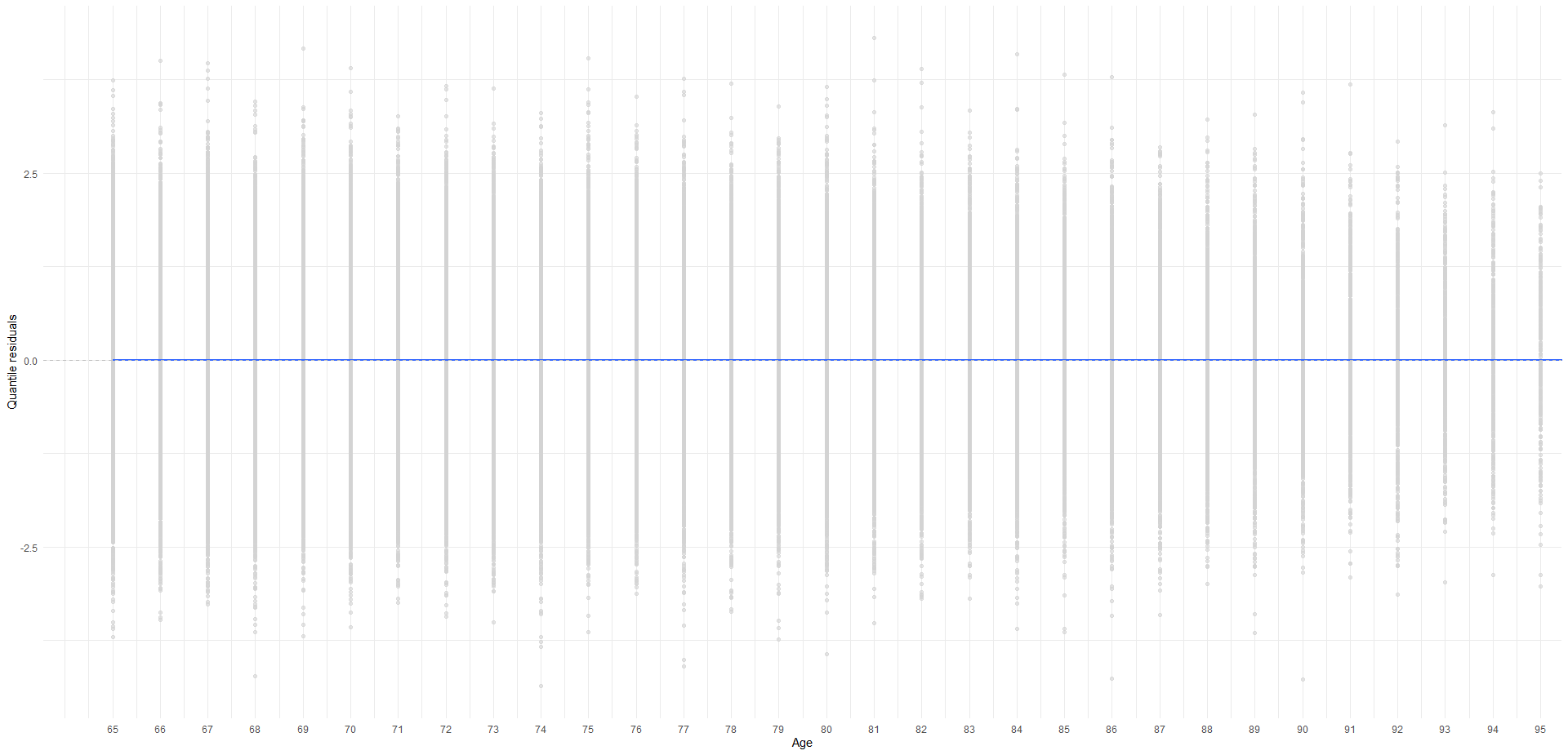


**Figure S4. Quantile residuals for age from the multivariable logistic regression model.**


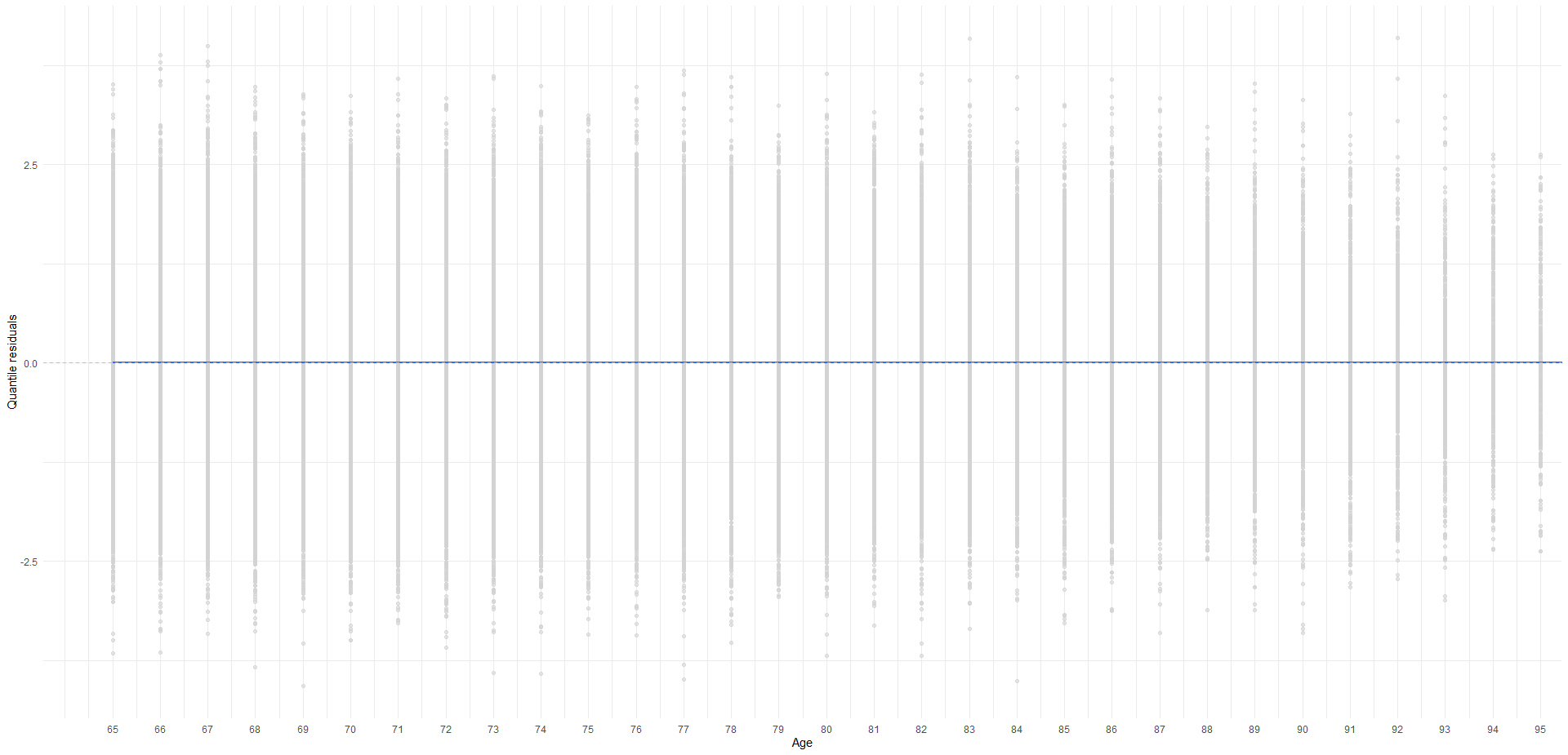


**Figure S5. The proportion of people who had a fall versus age. A linear regression line has been fitted to the plot.**


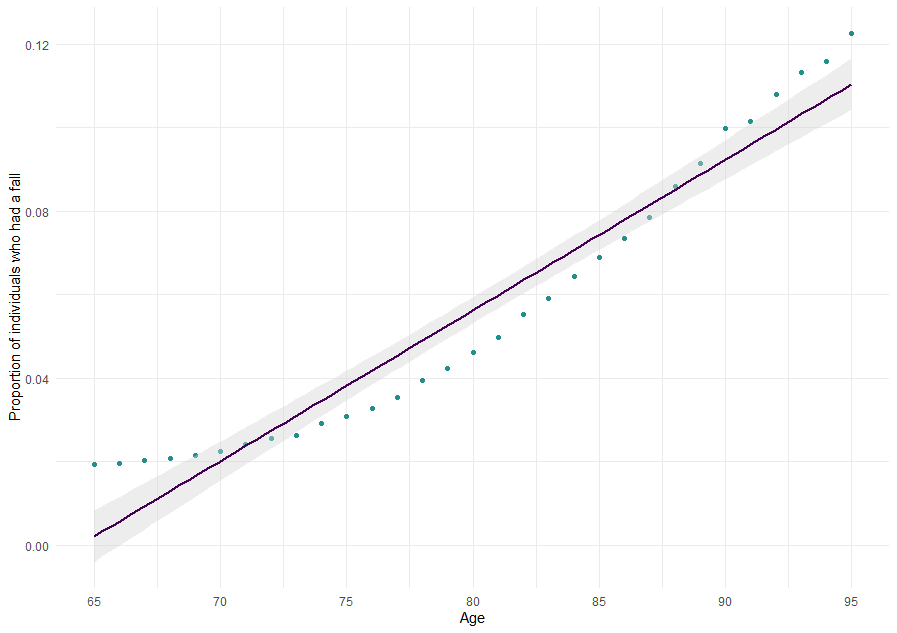


**Figure S6. The study year residuals for the null (intercept only) and multivariate multilevel logistic regression models. (a) The null model residuals ordered by the study year. (b) The null model ordered by the residual value. (c) The multivariate model residuals ordered by the study year. (d) The multivariate model ordered by the residual values.**


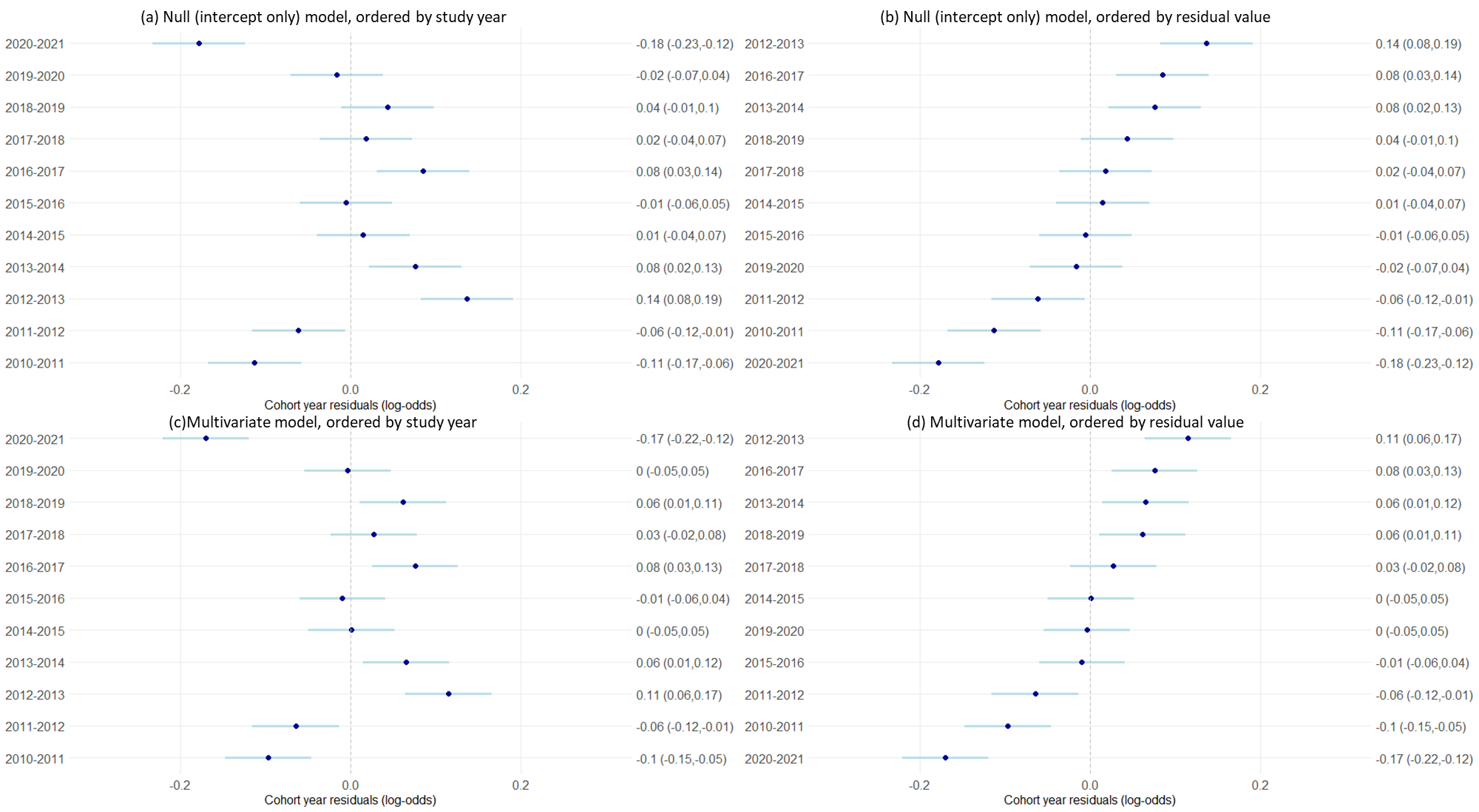


**Table S4. Multilevel logistic regression models for falls admissions resulting in a hospital or emergency department admission. The models include a random effect at the person level.**

| **Variables** | Null model |  |  |  |  |  |  |  |
| --- | --- | --- | --- | --- | --- | --- | --- | --- |
| Intercept | 0.04 (0.04,0.04) | 0 (0,0) | 0.049 (0.049,0.05) | 0.035 (0.035,0.036) | 0.021 (0.021,0.021) | 0.053 (0.052,0.053) | 0.038 (0.038,0.038) | 0.001 (0.001,0.001) |
| Age |  | 1.078 (1.078,1.079) |  |  |  |  |  | 1.05 (1.049,1.051) |
| Gender:Male |  |  | 0.6 (0.593,0.606) |  |  |  |  | 0.72 (0.712,0.728) |
| Dementia |  |  |  | 4.234 (4.162,4.307) |  |  |  | 2.156 (2.12,2.193) |
| eFI: Mild |  |  |  |  | 2.058 (2.033,2.083) |  |  | 1.579 (1.56,1.599) |
| eFI:Moderate |  |  |  |  | 3.774 (3.72,3.828) |  |  | 2.2 (2.167,2.234) |
| eFI:Severe |  |  |  |  | 6.121 (5.992,6.253) |  |  | 2.845 (2.781,2.909) |
| WIMD:2 |  |  |  |  |  | 0.889 (0.874,0.905) |  | 0.883 (0.869,0.897) |
| WIMD:3 |  |  |  |  |  | 0.676 (0.664,0.688) |  | 0.687 (0.676,0.699) |
| WIMD:4 |  |  |  |  |  | 0.596 (0.586,0.607) |  | 0.612 (0.602,0.623) |
| WIMD:5 Least Deprived |  |  |  |  |  | 0.704 (0.692,0.716) |  | 0.738 (0.726,0.75) |
| Previous fall |  |  |  |  |  |  | 1.865 (1.826,1.906) | 1.456 (1.427,1.485) |
|  |  |  |  |  |  |  |  |  |
| Random effects: individual level | 1.6936 | 1.37545 | 1.611 | 1.52293 | 1.16342 | 1.63991 | 1.32706 | 0.83881 |
| Standard error | 0.00908 | 0.00841 | 0.00892 | 0.00889 | 0.00812 | 0.00899 | 0.00858 | 0.00739 |

**Table S5. Multilevel multivariable logistic regression model with data from 2020 removed. The model includes a random intercept term for each year.**

| Observations: 4631573 |  |
| --- | --- |
| **The fixed part estimates:** | **Odds Ratio (95% CI)** |
| Intercept | 0.001 (0.001,0.001) |
| Age | 1.046 (1.045,1.047) |
| Gender:Male | 0.731 (0.723,0.738) |
| Dementia:Yes | 2.032 (2.001,2.064) |
| eFI (Reference: Fit) |  |
| Mild | 1.593 (1.573,1.613) |
| Moderate | 2.236 (2.203,2.268) |
| Severe | 2.923 (2.863,2.984) |
| WIMD (Reference: 1. Most Deprived) | |
| 2 | 0.889 (0.876,0.902) |
| 3 | 0.699 (0.688,0.71) |
| 4 | 0.626 (0.616,0.637) |
| 5. Least deprived | 0.759 (0.748,0.771) |
| Previous fall: Yes | 2.543 (2.5,2.586) |
| **Random effects (Year)** | **Coefficient (Standard Error)** |
| Intercept | 0.00373 (0.0017) |

**Table S6. Logistic regression model with an interaction term between year and dementia diagnosis included.**

| **Interaction model** | Odds Ratio | lower CI | upper CI |
| --- | --- | --- | --- |
| (Intercept) | 0.904 | 0.903 | 0.906 |
| Age | 1.002 | 1.002 | 1.002 |
| Gender (Male) | 0.989 | 0.989 | 0.99 |
| Dementia (Yes) | 1.055 | 1.052 | 1.058 |
| Year: 2011 | 1.001 | 1 | 1.001 |
| Year: 2012 | 1.006 | 1.005 | 1.007 |
| Year: 2013 | 1.005 | 1.005 | 1.006 |
| Year: 2014 | 1.003 | 1.003 | 1.004 |
| Year: 2015 | 1.003 | 1.002 | 1.004 |
| Year: 2016 | 1.005 | 1.005 | 1.006 |
| Year: 2017 | 1.004 | 1.003 | 1.005 |
| Year: 2018 | 1.006 | 1.005 | 1.006 |
| Year: 2019 | 1.003 | 1.002 | 1.004 |
| Year: 2020 | 0.998 | 0.997 | 0.998 |
| eFI: Mild | 1.01 | 1.01 | 1.011 |
| eFI: Moderate | 1.031 | 1.03 | 1.031 |
| eFI: Severe | 1.063 | 1.062 | 1.064 |
| WIMD: 2 | 0.995 | 0.995 | 0.996 |
| WIMD: 3 | 0.987 | 0.987 | 0.988 |
| WIMD: 4 | 0.984 | 0.983 | 0.984 |
| WIMD: 5.least deprived | 0.989 | 0.989 | 0.99 |
| Previous fall (Yes) | 1.078 | 1.077 | 1.079 |
| Dementia(Yes) x Year: 2011 | 1.011 | 1.007 | 1.015 |
| Dementia(Yes) x Year: 2012 | 1.036 | 1.032 | 1.041 |
| Dementia(Yes) x Year: 2013 | 1.012 | 1.008 | 1.017 |
| Dementia(Yes) x Year: 2014 | 1.004 | 1 | 1.009 |
| Dementia(Yes) x Year: 2015 | 1.002 | 0.998 | 1.006 |
| Dementia(Yes) x Year: 2016 | 1.02 | 1.016 | 1.024 |
| Dementia(Yes) x Year: 2017 | 1.005 | 1.001 | 1.009 |
| Dementia(Yes) x Year: 2018 | 0.998 | 0.994 | 1.002 |
| Dementia(Yes) x Year: 2019 | 0.998 | 0.994 | 1.002 |
| Dementia(Yes) x Year: 2020 | 0.994 | 0.99 | 0.998 |

**Falls coding**

*Patient Episode Database for Wales ICD10 codes:*

| **ICD10** | **Description** |
| --- | --- |
| W01 | Fall on same level from slipping, tripping and stumbling |
| W03 | Other fall on same level due to collision with, or pushing by, another person |
| W04 | Fall while being carried or supported by other persons |
| W05 | Fall involving wheelchair |
| W06 | Fall involving bed |
| W07 | Fall involving chair |
| W08 | Fall involving other furniture |
| W10 | Fall on and from stairs and steps |
| W11 | Fall on and from ladder |
| W12 | Fall on and from scaffolding |
| W13 | Fall from, out of or through building or structure |
| W17 | Other fall from one level to another |
| W18 | Other fall on same level |
| W19 | Unspecified fall |

*Emergency Department DataSet http://www.datadictionary.wales.nhs.uk/#!WordDocuments/mechanismofinjury.htm*

Mechanism of injury: 01 - fall/trip/slip
